# Compression wraps as adjuvant therapy in the management of acute systolic heart failure

**DOI:** 10.1016/j.heliyon.2023.e19008

**Published:** 2023-08-07

**Authors:** Raef Ali Fadel, Renato Cerna Viacava, Tarek Makki, Carina Dagher Fadel, Kelly Malette, Zachary D. Demertzis, Guneet Ahluwalia, Joseph Miller, Cori Russell

**Affiliations:** a Henry Ford Health System, Division of Cardiovascular Medicine, Detroit, MI, USA; b Henry Ford Health System, Department of Internal Medicine, Detroit, MI, USA; c Beaumont Health System, Department of Cardiovascular Medicine, Dearborn, MI, USA; d Beaumont Health System, Department of Cardiovascular Medicine, Royal Oak, MI, USA; e Saint Joseph Mercy Health System, Department of Cardiology, Pontiac, MI, USA; f Henry Ford Health System, Emergency Medicine, Detroit, MI, USA

**Keywords:** Heart failure, Compression wraps, Edema

## Abstract

**Background:**

Guidelines recommend targeting decongestion in management of decompensated HF, with lower extremity edema often serving as the clinical target. LECW are seldom used in the acute setting, with a paucity of data on efficacy in HF, despite serving as a cornerstone of chronic lymphedema management.

**Primary objective:**

Study the efficacy and safety of LECW in acute decompensated HF.

**Methods:**

Open-label, randomized, parallel-group clinical trial.

**Primary outcomes:**

Days on intravenous (IV) diuretic therapy, total hospital length of stay (LOS), and 30-day all-cause readmission.

**Results:**

32 patients were enrolled, with 29 patients completing the study. Enrollment was suspended due to the COVID-19 pandemic. Overall LOS was shorter in the intervention group (3.5 vs 6 days, p = 0.05), with no significant difference in total days on IV diuresis or 30-day readmission rate with use of LECW. Fewer patients required continuous diuretic infusion after treatment with LECW (0 vs 7 patients, p = 0.027). The intervention group scored significantly better on the MLWHF (55.5 vs 65, p = 0.021), including both the physical and emotional dimension scores. No adverse events were reported with use of LECW, including a significantly lower incidence of AKI (1 vs 13 patients, p = 0.005).

**Conclusion:**

The use of LECW resulted in reduced hospital LOS compared to standard therapy, with no difference in days of IV diuresis administration or 30-day readmission. Treatment with LECW also resulted in less continuous IV diuretic therapy, fewer incidence of AKI, and improved quality of life. Trends toward less escalation of diuresis, and greater reduction in edema were also observed.

## Nomenclature

**Abbreviations**
LOS: length of stay
IV: intravenous
PO: oral
RN: registered nurse
LECW: lower extremity compression wrap
HFrEF: heart failure with reduced ejection fraction
CKD: chronic kidney disease
AKI: acute kidney injury
BNP: brain natriuretic peptide
MLWHF: Minnesota Living With Heart Failure
QOL: quality of life
NYHA: New York Heart Association
DVT: deep vein thrombosis
PE: pulmonary embolism
COVID19: coronavirus disease 2019

## 1 Introduction

Heart failure (HF) is a complex syndrome, leading to significant morbidity and mortality of afflicted patients, and currently accounting for over 10% of total healthcare expenditure in the United States [1]. Such high prevalence has garnered much of the medical community's attention in basic science and clinic research, and as a result the life expectancy of patients with HF has increased dramatically over the past few decades [2]. As a result, patients living with HF suffer from the symptoms longer, resulting in increased emergency room visits and hospitalizations [1,2].

Of the many symptoms affecting patients with HF, lower extremity edema remains among the most prevalent and debilitating. In one review of over 4000 patients with diagnosed HF, ankle edema was present in roughly 87% of cases [3]. Current guidelines from the American College of Cardiology/American Heart Association (ACC/AHA) recommend targeting overall decongestion in management of patients with decompensated HF [4], and with the prevalence of lower extremity edema in admitted patients, management is often targeted at clinical edema reduction.

Despite serving a cornerstone role in management of chronic lymphedema, lower extremity compression wraps (LECW) have limited evidence in the management of HF [[5], [6], [7]]. There are also no guidelines for the management of lower extremity edema with compression wraps in patients with HF [4,8]. Independent reviews have suggested a potential harm with use of compression therapy in patients with HF, particularly as it relates to hemodynamics and pulmonary edema [7,9,10], with other reviews demonstrating neutral [11], or positive [6,12], impacts.

In the Compression Wraps as Adjuvant Therapy in the Management of Acute Systolic Heart Failure (CATAS HF) trial, we evaluated the efficacy of LECW in treatment of HF with reduced ejection fraction (HFrEF), hypothesizing that use of LECW would lead to symptomatic improvement and reduced length of stay.

## 2 Methods

### 2.1 Trial design and oversight

This was a single-center, randomized, open-label, parallel group clinical trial evaluating patients admitted to the telemetry unit of a tertiary comprehensive care hospital in Detroit, Michigan. This investigation was approved by an institutional review board (IRB #12985). Informed consent was obtained from all participants in the study prior to enrollment. This was a non-sponsored, non-funded study. The clinicaltrials.gov registration identifier is NCT04095416.

The investigation committee developed and amended the protocol and had scientific oversight on the development of the statistical analysis plan, recruitment of patients, the quality of follow-up, and the procurement and analysis of data. The research coordinators formed an independent data and safety monitoring committee and were responsible for reviewing application of LECW as well as evaluating for any evidence of skin breakdown, pressure wounds, or trauma. The safety committee was also responsible for evaluating data at pre-specified junctions to ensure adherence to safety endpoints. The statistical analyses were performed by employees of the Public Health Sciences department of the research site, with the oversight of the trial leadership. The first author, who had unrestricted access to the data, prepared the first draft of the manuscript, which was then reviewed and edited by all the authors. The authors made the decision to submit the manuscript for publication, assume full responsibility for the accuracy and completeness of the analyses, and attest to the fidelity of the trial to the protocol and the statistical analysis plan.

### 2.2 Patient inclusion, exclusion, and withdrawal

Adult patients age 18 years or older with a history of chronic HF (functional class II, III, or IV) with a left ventricular ejection fraction of 40% or less as determined by echocardiography within 6 months of evaluation, were eligible for enrollment in the trial. Final inclusion criteria required admission to the telemetry unit for intravenous (IV) diuretic therapy in the management of acute decompensated HF, and presence of lower extremity pitting edema on physical examination.

Patients were excluded if any of the following criteria were met: an active diagnosis of chronic kidney disease (CKD) stage 5 or on hemodialysis; peripheral neuropathy; active infection (bacterial, viral, or parasitic) or skin breakdown (any stage pressure ulcer); a history of severe peripheral arterial disease as defined by presence of a Ankle-Brachial Index (ABI) value of <0.4 or severe symptoms; history of deep vein thrombosis (DVT) or pulmonary embolism (PE); or intolerance to IV diuresis for any reason.

Patients were withdrawn from the trial if they could not tolerate application of LECW, evidence of allergic reaction to the wrap material, evidence of wounds or skin breakdown after application, or removal of the LECW for over 12 consecutive hours across a 48-h period, or over 6 consecutive hours in a 24-h period.

### 2.3 Randomization, intervention, and follow-up

After inclusion, patients underwent 2:1 randomization through a computer software into the control and intervention arms, respectively.

Patients in the intervention arm underwent application of lower extremity compression wraps by trained research personnel or registered nurses (RN). Instructions for the application of LECW can be found in the supplemental section (S2,3 in supplementary), developed by the research coordinators [13]. LECW were applied to maximum tolerated compression, as determined by patient report and symptoms of discomfort. Patients were educated on the application of the wraps and instructed to limit removal of wraps except for the following: nurse or research team assessment for wounds, daily hygiene, and primary treatment team clinical assessment. If wraps were removed, they were promptly replaced by the research team or RN.

All patients enrolled in the trial received standard of care for management of decompensated HF. Each patient completed the Minnesota Living with Heart Failure (MLWHF) questionnaire at the end of their hospital stay, prior to discharge.

The follow-up period was 30-days after discharge from the hospital, through telecommunication between the research team and enrolled patients. If no response was received from the patient, chart review was used to detect and re-admission to the treatment institution or surrounding hospitals affiliated with the electronic health record.

### 2.4 Primary, secondary, and safety endpoints

The primary end points of this study included: total hospital length of stay (LOS), total days of IV diuretic therapy, and 30-day all-cause hospital readmission.

The secondary outcomes included Discharge serum brain natriuretic peptide (BNP) level, reduction in serum BNP level, net fluid change, percent weight reduction, and quality of life as assessed by the MLWHF questionnaire.

Safety endpoints included incidence of acute kidney injury (AKI), lower extremity wounds or pressure ulcers, ischemic injury, and escalation to the intensive care unit.

### 2.5 Study definitions

AKI was assessed in accordance with the kidney disease: Improving Global Outcomes (KDIGO) 2012 practice guidelines [14]. We defined pitting edema presence and degree using depth of indentation and rebound time, with a score of 0 indicating no clinical edema, and a score of 4+ indicating greater than 6 mm indentation lasting longer than 2 min before rebound [15]. Continuous IV diuresis therapy was defined as the use of diuretics in an intravenous formulation infused at a continuous rate for greater than 4 h, and escalation of diuresis was defined as an increase in the potency, dosing, or frequency of administered diuretic. Adjuvant diuretic therapy was defined as the addition of an oral (PO) or IV diuretic to the primary treatment regimen after admission to the telemetry unit, with at least two doses administered in a 24-h period.

Decompensation of HF was defined using clinical signs (jugular venous distension, lower extremity edema, ascites, S3 gallop), serum biomarkers (BNP >150 pg/mL), or radiographic evidence (cardiomegaly, pulmonary edema) of congestion. Diagnosis was left to the discretion of the treating physician. Diagnosis was confirmed by the research team for enrollment, including confirming presence of lower extremity edema and BNP elevation.

HF quality of life (QOL) was assessed with the MLWHF questionnaire, the most widely adopted, disease specific tool, well validated for assessment of quality of life in patients with HF as it relates to treatment [16,17]. Degrees of change in the MLWHF scores were classified as significant (by a change ≥5 units) or not changed (change <5 units) based on prior studies [18].

### 2.6 Statistical analysis

The initial protocol design called for 120 patients to be enrolled, including 60 patients in the intervention arm, and 60 patients in the standard care arm. However, as a result of premature termination of the trial due to the COVID-19 pandemic, we instead performed a post-hoc analysis on the patients who had already completed the trial, and determined that a target number of 36 patients would detect a significant difference in all primary endpoints at the pre-stated enrollment ratio of 2:1. Assuming a national mean length of stay of 5 days for HF admissions [19], 36 patients would provide a power of 80% to detect a difference of 1 hospital day, as well as a difference of 1 day of IV diuretic therapy, at a two-sided alpha level of 0.05. This target enrollment also provided similar power to detect a net change of 1+ in clinical lower extremity edema, as well as a 15% decrease in net MLWHF score and in the physical and emotional dimension scores, also at a two-sided alpha level of 0.05.

Data analysis was conducted through the Public Health Sciences department of the treatment institution and included descriptive statistics and univariate comparisons between treatment groups. Continuous variables were described as mean (standard deviation), and categorical variables as frequency rates (n) and percentages (%). The Mann-Whitney U or *t*-test were used for continuous variables, whereas chi-squared or Fisher's exact test were used for categorical variables. A two-sided α < 0.05 was considered statistically significant. Poisson regression model was used to examine the effect of LECW on hospital LOS.

## 3 Results

From August 2019 to March 2020, a total of 46 patients were evaluated for enrollment, with 32 patients (69.6%) meeting inclusion criteria and randomly assigned to receive either LECW in addition to standard care (13 patients), or standard care alone (19 patients); 14 patients (30.4%) were excluded from the study; 29 patients completed the study, with 3 patients in the intervention group withdrawing early for intolerance or noncompliance (Fig. S1 in supplementary). Due to the coronavirus disease 2019 (COVID-19) pandemic, the study was halted March 11, 2020 and enrollment was discontinued prior to achieving our target enrollment The median follow-up was 30 days, with all 29 patients completing follow-up.

The baseline characteristics of patients in the two trial groups were similar (Table 1). Over 75% of patients were male, and over 65% were black. Underlying etiology of HF was ischemic in 31% of patients, as compared to 58.6 non-ischemic, with all included patients meeting New York Heart Association (NYHA) class 3 or worse. Degree of lower extremity edema was also similar, with 58.6% of patients presenting with 3+ pitting edema or worse. Approximately 24.1% of all patients had evidence of AKI on presentation, and 34.5% had an estimated GFR of less than 60 ml per minute per 1.73 m^2^, with no statistically significant difference between the two groups.

**Table 1:** Comparisons of baseline characteristics of the Control and Intervention groups.

	Overall	Control	Intervention	p
n	29	19	10	
Age - years (mean (SD))	64.2 (13.4)	64.5 (12.0)	63.7 (16.4)	0.878
Ejection fraction baseline - % (mean (SD))	28.1 (9.5)	26.05 (8.7)	32.0 (10.3)	0.112
Admission BNP - pg/mL (mean (SD))	2095 (1391)	1997 (1296)	2279 (1613)	0.613
Admission weight - kg (mean (SD))	93.7 (24.7)	91.2 (21.3)	98.3 (30.9)	0.471
Baseline Creatinine - mg/dL (mean (SD))	1.24 (0.54)	1.26 (0.57)	1.20 (0.52)	0.801
Albumin on admit - g/dL (mean (SD))	3.4 (0.5)	3.4 (0.4)	3.2 (0.6)	0.239
Oxygen on admit - L (mean (SD))	1 (1)	1 (1)	1 (1)	0.084
Sodium admit - mg/dL (mean (SD))	136 (4.8)	136 (3.6)	136 (6.9)	0.899
Potassium admit - mg/dL (mean (SD))	4.1 (0.5)	4.1 (0.5)	4.2 (0.3)	0.467
Chloride admit - mEq/L (mean (SD))	101.9 (5.0)	102.1 (4.0)	101.5 (6.8)	0.783
BUN admit - mg/dL (mean (SD))	29.5 (21.3)	32.2 (24.1)	24.4 (14.3)	0.360
Creatinine admit - mg/dL (mean (SD))	1.47 (0.81)	1.60 (0.89)	1.25 (0.60)	0.277
Magnesium admit - mg/dL (mean (SD))	1.9 (0.3)	2.0 (0.3)	1.7 (0.2)	**0.024**
Phosphorus admit - mg/dL (mean (SD))	3.8 (0.7)	3.7 (0.8)	3.8 (0.3)	0.771
Admit SBP - mmHg (mean (SD))	135 (27)	132 (29)	142 (23)	0.353
Admit DBP - mmHg (mean (SD))	85 (20)	86 (23)	82 (15)	0.678
Admit MAP - mmHg (mean (SD))	101 (21)	101 (23)	102 (15)	0.891
Females (n (%))	7 (24.1)	4 (21.1)	3 (30.0)	0.665
Black race (n (%))	22 (75.9)	13 (68.4)	9 (90.0)	0.367
Heart failure etiology (n (%))				0.441
unknown	2 (6.9)	2 (10.5)	0 (0.0)	
ischemic	9 (31.0)	7 (36.8)	2 (20.0)	
non-ischemic	17 (58.6)	9 (47.4)	8 (80.0)	
other	1 (3.4)	1 (5.3)	0 (0.0)	
NYHA class on admit (n (%))				0.126
2	2 (6.9)	0 (0.0)	2 (20.0)	
3	9 (31.0)	7 (36.8)	2 (20.0)	
4	18 (62.1)	12 (63.2)	6 (60.0)	
Valvular disease history (n (%))	12 (41.4)	9 (47.4)	3 (30.0)	0.449
Mitral Regurgitation	9 (31.0)	6 (31.6)	3 (30.0)	1
Tricuspid Regurgitation	7 (24.1)	7 (36.8)	0 (0.0)	0.063
Aortic Regurgitation	2 (6.9)	1 (5.3)	1 (10.0)	
Aortic Insufficiency	2 (6.9)	1 (5.3)	1 (10.0)	
Arrhythmia history (n (%))	12 (41.4)	8 (42.1)	4 (40.0)	1
Diabetes mellitus (n (%))	17 (58.6)	12 (63.2)	5 (50.0)	0.694
Hypertension (n (%))	20 (69.0)	13 (68.4)	7 (70.0)	1
Obstructive sleep apnea (n (%))	2 (6.9)	2 (10.5)	0 (0.0)	0.532
Chronic obstructive pulmonary disease (n (%))	8 (27.6)	6 (31.6)	2 (20.0)	0.675
Coronary artery disease (n (%)	11 (37.9)	8 (42.1)	3 (30.0)	0.694
Chronic kidney disease (n (%))	14 (48.3)	10 (52.6)	4 (40.0)	0.701
Chronic kidney disease stage (n (%))				
≤1	15 (51.7)	9 (47.4)	6 (60.0)	
2	4 (13.8)	3 (15.8)	1 (10.0)	
3	6 (20.7)	4 (21.1)	2 (20.0)	
4	4 (13.8)	3 (15.8)	1 (10.0)	
Degree edema on admit (n (%))				0.171
1+	6 (20.7)	4 (21.1)	2 (20.0)	
2+	6 (20.7)	6 (31.6)	0 (0.0)	
3+	14 (48.3)	8 (42.1)	6 (60.0)	
4+	3 (10.3)	1 (5.3)	2 (20.0)	

BNP: brain natriuretic peptide; on admit: on admission to the hospital; BUN: blood urea nitrogen; SBP: systolic blood pressure; DBP: diastolic blood pressure; MAP: mean arterial pressure; NYHA: New York Heart Association; mg: milligram; dL: deciliter; L: liter; mEq: milliequivalent.

Bolded p-values indicate statistically significant values < 0.05.

Patients in the intervention arm maintained LECW application for 80.2% (SD 3) of their treatment period. A total of 3 patients did not complete the study in the intervention arm (all due to non-compliance with LECW use), with all patients completing the study in the control group. No patients in the intervention arm experienced skin infections, allergic reactions, or arterial/venous complications of LECW use.

There was a reduction in the use of continuous IV diuretic therapy in the intervention group as compared to control (0 vs 7 patients, p = 0.028). There was no significant difference in escalation of IV diuretic therapy (0 vs 5 patients, p = 0.079), or use of adjuvant diuresis while inpatient (0 vs 3 patients, p = 0.198) in patients treated with LECW compared to standard of care. There was no significant difference in the type of oral diuretic therapy on discharge (Table 2).

**Table 2:** LECW and medication management of heart failure during admission and at discharge for Control and Intervention groups.

	Overall	Control	Intervention	p
n	29	19	10	
Time LECW applied - hours (mean (SD))		0 (0)	89 (49)	
% time with LECW applied (mean (SD))		0 (0)	80 (3)	
IV Diuretic type escalated (n (%))	5 (17.2)	5 (26.3)	0 (0.0)	0.079
IV Diuretic continuous infusion used (n (%))	7 (24.1)	7 (36.8)	0 (0.0)	**0.028**
Adjuvant diuretic used (n (%))	3 (10.3)	3 (15.8)	0 (0)	0.198
Daily dose of IV diuresis – mg (median (IQR))^+^	160 (80–160)	140 (80–160)	160 (80–160)	0.789
Total IV diuresis - mg (median (IQR))^+^	480 (400–800)	440 (400–800)	480 (380–420)	0.801
PO Diuretic type on discharge (n (%))				0.366
Furosemide	15 (51.7)	11 (57.9)	4 (40.0)	
Bumetanide	12 (41.4)	7 (36.8)	5 (50.0)	
Torsemide	1 (3.4)	0 (0.0)	1 (10.0)	
Hydrochlorothiazide	0 (0.0)	0 (0.0)	0 (0.0)	
Spironolactone on discharge (n (%))	14 (48.3)	7 (36.8)	7 (70.0)	0.096

LECW: lower extremity compression wraps; IV: intravenous; PO: oral; mg: milligrams.

+Reported in furosemide equivalent doses.

Bolded p-values indicate statistically significant values < 0.05.

### 3.1 Primary outcomes

There was no significant difference in total days of IV diuresis administration between patients treated with LECW as compared to standard care (5.0 [1.8] vs 4.2 [3.1] days, p = 0.372).

An overall decrease in LOS was observed in the intervention group as compared to control (3.5 [3–7] vs 6 [5–10] days, p = 0.05). A Poisson regression model was used to examine the effect of intervention on LOS (IRR = 0.62, 95% CI 0.44–0.86, p = 0.005), suggesting an overall 38% shorter LOS in the intervention group.

There was no significant difference in 30-day all cause readmission rates, including readmission specifically for HF symptoms (Table 3).

**Table 3:** Outcomes data for intervention and control groups.

	Overall	Control	Intervention	
n	29	19	10	
**Primary Outcomes**				
Total days of IV diuresis (mean (SD))	4.7 (2.3)	5.0 (1.8)	4.2 (3.1)	0.372
Length of stay - days (median [IQR])	6.0 [4.0, 8.0]	6.0 [5.0, 9.5]	3.5 [3.0, 5.5]	**0.022**
30-day all-cause readmission (n (%))	7 (24.1)	5 (26.3)	2 (20.0)	0.999
30-day readmission for CHF (n (%))	5 (17.2)	4 (21.1)	1 (10.0)	0.633
**Secondary Outcomes**				
AKI during admit (n (%))	14 (48.3)	13 (68.4)	1 (10.0)	**0.005**
Required dialysis inpatient (n (%))	0 (0)	0 (0)	0 (0)	0.999
Discharge BNP - pg/mL (mean (SD))	1295 (1091)	1238 (896)	1404 (1441)	0.705
% BNP change (mean (SD))	−38.1 (28.8)	−39.2 (27.6)	−38.1 (22.8)	0.620
Net intake/output - L (mean (SD))	−7.44 (6.78)	−7.08 (6.21)	−8.13 (8.05)	0.701
Weight change - % (mean (SD))	−7.2 (11.3)	−7.60 (10.0)	−5.51 (7.3)	0.320
Net weight change - kg (mean (SD))	6.3 (5.2)	6.5 (5.5)	5.8 (4.8)	0.727
MLWHF Score - overall (mean (SD))	63.2 (11.8)	66.7 (12.3)	56.4 (7.0)	**0.021**
Physical dimension score (mean (SD))	21.2 (4.0)	23.1 (3.2)	17.7 (2.8)	**<0.001**
Emotional dimension score (mean (SD))	9.2 (4.0)	11.1 (3.2)	5.7 (2.8)	**<0.001**
**Safety Endpoints**				
Development of pressure wounds (n (%))	0	0 (0)	0 (0)	
Development of pressure ulcers (n (%))	0	0 (0)	0 (0)	
Development of ischemic injury (n (%))	0	0 (0)	0 (0)	
Escalation to ICU level care	1 (4.0)	1 (5.3)	0 (0)	

BNP: brain natriuretic peptide; on admit: on admission to the hospital; BUN: blood urea nitrogen; SBP: systolic blood pressure; DBP: diastolic blood pressure; MAP: mean arterial pressure; NYHA: New York Heart Association; mg: milligram; dL: deciliter; L: liter; mEq: milliequivalent.

Bolded p-values indicate statistically significant values < 0.05.

### 3.2 Secondary outcomes

Discharge BNP levels were similar between the two groups (1404 vs 1238, p = 0.705), with no statistical significance in percent weight change, despite a large percent reduction in the intervention group as compared to control (42.8% [22.8] vs 23.5% [57.6], p = 0.320). Similarly, there was no significant difference in net intake and output, or in weight change between the two groups across the hospital stay (Table 3).

HF QOL, as assessed by the MLWHF questionnaire, was significantly lower in the intervention group, with reductions in net score (55.5 vs 65, p = 0.021), physical dimension score (17.5 vs 23, p < 0.001), and emotional dimension score (5.5 vs 11, p < 0.001).

There was a higher rate of AKI in the control group, with 13 patients experiencing an AKI as compared to only 1 patient in the intervention group (p = 0.005). There were no reported adverse events with use of LECW (Table 3).

Patients receiving LECW experienced decrease in severity of lower extremity pitting edema compared to controls, trending toward statistical significance (1.5+ [1–2] vs 1+ [1–2], p = 0.072). There was no significant difference in the levels of sodium, potassium, magnesium, or chloride change across the hospitalization (Supplementary Table 1).

## 4 Discussion

In this single-center, randomized, open-label, parallel group clinical trial evaluating patients admitted to the telemetry unit of a tertiary comprehensive care hospital for management of HF with reduced ejection fraction, the use of LECW resulted in a significant difference in the primary outcome of hospital length of stay, but did not result in a significant difference in the primary outcome of total days of IV diuresis or 30-day all-cause readmission. Additionally, patients treated with LECW received less continuous IV diuresis, and experienced lower incidence of AKI, with improvement in MLWHF QOL score (including both physical and emotional indices). Trends toward fewer total days of IV diuresis, less escalation of diuresis, and greater reduction in edema were also observed.

Our study represents the first clinical evaluation of compression wrap use in this patient population and suggests clinical applicability of LECW as a low cost, simple adjuvant therapy. With no observed adverse clinical outcomes, our data instead suggests multiple potential therapeutic benefits for clinical use of LECW, most importantly reduction in use of continuous diuretic therapy, fewer AKI rates, and shorter hospital LOS. Additionally, patient assessed HF QOL was improved as compared to control. These results were demonstrated in patients with predominantly NYHA class 3–4 HF, and varying CKD stages, contrasting previous reports of potential harm with use of lower extremity compression therapy [7,9,10].

The difference in rates of AKI between the intervention and control groups is one of clinical significance. The incidence of AKI in patients hospitalized with decompensated HF is well described, as is the association of worsening renal function with adverse outcomes [[20], [21], [22]]. Several meta-analyses demonstrated increased mortality and rate of hospitalization associated with worsening renal function in patients with HF [23,24]. With no significant difference in prevalence of CKD between the two groups, fewer rates of AKI with use of LECW can be multifactorial, resulting from redistribution of fluid compartments to augment intravascular volume, as evidenced by a trend toward statistical significance in degree of edema; also, the potential to decrease provider use of diuresis and avoiding complications of hypovolemia, highlighted by a trend toward reduction in diuretic escalation, use of continuous diuresis, and adjuvant diuretic use.

The reduction of hospital LOS by 38% with addition of LECW to standard inpatient management in this study was encouraging, with the potential to impact care of patients with acute HF exacerbations. Considering the significant impact of recurrent hospitalizations in this patient population, along with associated morbidity, reduced LOS can result in improved QOL [1,2]. Accordingly, patients treated with LECW demonstrated an average overall improvement of 10.3 points as compared to control, with improvement of 5.4 points in both the physical and emotional indices. Such improvements in QOL were demonstrated in the Val-HeFT trial [18], as a result of valsartan addition to standard therapy, lending further credence to the use of LECW inpatient as adjuvant therapy in the management of patients with HF. It should be noted that, although we demonstrate a reduction in hospital LOS in patients treated with LECW, the primary cause remains unclear. In regards to augmentation of diuresis, there was no significant difference in degree of edema improvement, total diuresis, net fluid loss, or weight, and therefore none of these endpoints explain why the patients were discharged sooner than those in the standard care arm. Whether the improvement in QOL factored into clinician decision making of readiness to discharge is one possibility. Additionally, the increased incidence in AKI could also impact length of stay, as providers may have felt it prudent to keep patients hospitalized until kidney function stabilized.

There are several limitations to discuss, and to generalize the results of this study to all patients with decompensated HF several of components of the trial must be addressed. First and foremost, the COVID-19 pandemic, which has affected the lives of millions world-wide to-date, first impacted the state of Michigan in March 2020, as the trial was ongoing. This resulted in the abrupt cessation of patient enrollment, and subsequent termination of the trial. On post-hoc analysis, the study achieved 75% power for all primary endpoints, however, was below target enrollment of 36 patients (18 patients per group). It is also important to highlight the variability in diuretic management of patients admitted with decompensated HF. Management was left to the discretion of the primary treatment team, who also decided when to transition to oral diuresis and to discharge patients, creating a potential confounder in the length of stay and overall satisfaction of patients. Controlling for this may limit confounding. Additionally, the protocolized application of LECW in this trial was coordinated with nursing staff, physician teams, and research coordinators, with significant patient education and re-education with maintenance of the wraps for extended periods of time. The practicality of such an approach may come into question in hospitals with limited resources or high patient turnover.

## 5 Conclusion

In this open-label randomized clinical trial of patients hospitalized for HFrEF, the use of LECW resulted in less continuous IV diuretic therapy, fewer incidence of AKI, improved physical and emotional HF QOL, and reduced hospital LOS. Trends toward fewer total days of IV diuresis, less escalation of diuresis, and greater reduction in edema were also observed. Further large-scale trials are necessary prior to incorporating LECW into standard medical care for patients admitted with decompensated HF.

## Funding

None.

## Previous publication/presentation

ESC Congress 2021.

## Author contribution statement

Dr. Raef Ali Fadel: conceived and designed the experiments, performed the experiments, analyzed and interpreted the data, and wrote the paper.

Drs. Cori Russell, Joseph Miller and Tarek Makki: conceived and designed the experiments; Wrote the paper.

Drs. Carina Dagher Fadel, Kelly Malette, Zachary Demertzis and Guneet Ahluwalia: Performed the experiments; Wrote the paper.

Dr. Renato Cerna Viacava: Analyzed and interpreted the data; wrote the paper.

## Data availability statement

The authors do not have permission to share data.

## Declaration of competing interest

The authors declare that they have no known competing financial interests or personal relationships that could have appeared to influence the work reported in this paper.

## Figures and Tables

**figs1:**
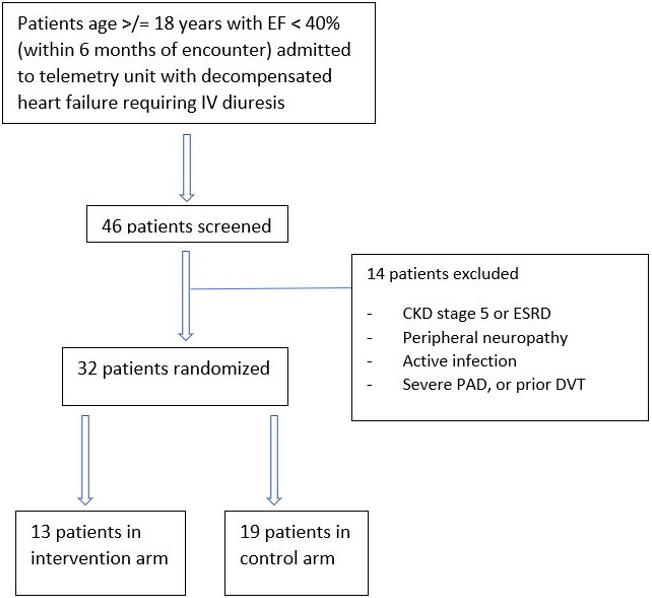


**figs2:**
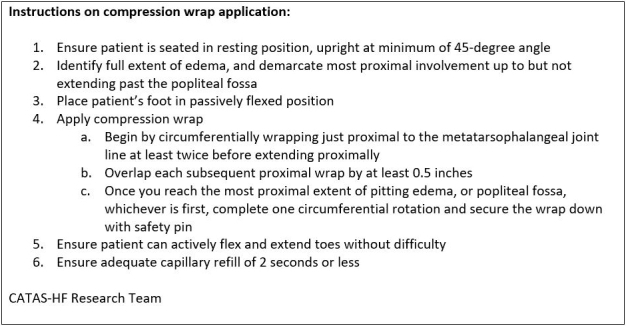


**figs3:**
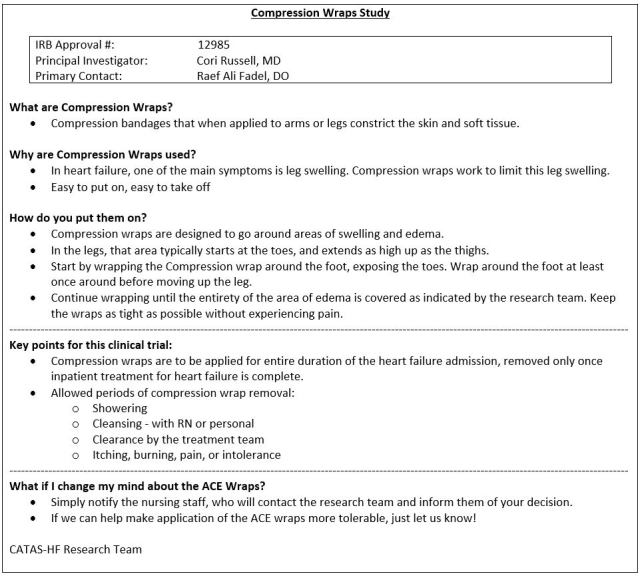


## References

[1] Mozaffarian D., Benjamin E.J., Go A.S. (2016). Heart disease and Stroke statistics-2016 Update: a report from the American heart association. Circulation.

[2] Alpert C.M., Smith M.A., Hummel S.L., Hummel E.K. (2017). Symptom burden in heart failure: assessment, impact on outcomes, and management. Heart Fail. Rev..

[3] Vijayakrishnan R., Steinhubl S.R., Ng K. (2014). Prevalence of heart failure signs and symptoms in a large primary care population identified through the use of text and data mining of the electronic health record. J. Card. Fail..

[4] Yancy C.W., Jessup M., Bozkurt B. (2017). 2017 ACC/AHA/HFSA Focused Update of the 2013 ACCF/AHA guideline for the management of heart failure: a report of the American College of Cardiology/American heart association Task Force on clinical practice guidelines and the heart failure Society of America. Circulation.

[5] Galm O., Jansen-Genzel W., von Helden J., Wienert V. (1996).

[6] Leduc O., Crasset V., Leleu C. (2011). Impact of manual lymphatic drainage on hemodynamic parameters in patients with heart failure and lower limb edema. Lymphology.

[7] Urbanek T., Juśko M., Kuczmik W.B. (2020). Compression therapy for leg oedema in patients with heart failure. ESC Heart Fail..

[8] Ponikowski P., Voors A.A., Anker S.D. (2016). 2016 ESC guidelines for the diagnosis and treatment of acute and chronic heart failure. Rev. Esp. Cardiol..

[9] Hirsch T. (2018). Oedema drainage and cardiac insufficiency—when is there a contraindication for compression and manual lymphatic drainage?. Phlebologie.

[10] Dereppe H., Hoylaerts M., Renard M., Leduc O., Bernard R., Leduc A. (1990). [Hemodynamic impact of pressotherapy]. J. Mal. Vasc..

[11] Bain R.J., Tan L.B., Murray R.G., Davies M.K., Littler W.A. (1989). Central haemodynamic changes during lower body positive pressure in patients with congestive cardiac failure. Cardiovasc. Res..

[12] Feldman A.M., Silver M.A., Francis G.S. (2006). Enhanced external counterpulsation improves exercise tolerance in patients with chronic heart failure. J. Am. Coll. Cardiol..

[13] Moffatt C. (2002). Four-layer bandaging: from concept to practice. Int. J. Low. Extrem. Wounds.

[14] (2012). Kidney disease: improving global outcomes (KDIGO) acute kidney injury work group. KDIGO clinical practice guideline for acute kidney injury. Kidney Inter..

[15] Brodovicz K.G., McNaughton K., Uemura N. (2009). Reliability and feasibility of methods to quantitatively assess peripheral edema. Clin. Med. Res..

[16] Garin O., Herdman M., Vilagut G. (2014). Assessing health-related quality of life in patients with heart failure: a systematic, standardized comparison of available measures. Heart Fail. Rev..

[17] Rector T.S., Cohn J.N. (1992). Assessment of patient outcome with the Minnesota Living with Heart Failure questionnaire: reliability and validity during a randomized, double-blind, placebo-controlled trial of pimobendan. Pimobendan Multicenter Research Group. Am. Heart J..

[18] Majani G., Giardini A., Opasich C. (2005). Effect of valsartan on quality of life when added to usual therapy for heart failure: results from the Valsartan Heart Failure Trial. J Card Fail..

[19] Samsky M.D., Ambrosy A.P., Youngson E. (2019). Trends in readmissions and length of stay for patients hospitalized with heart failure in Canada and the United States. JAMA Cardiol..

[20] Gottlieb S.S., Abraham W., Butler J., Forman D.E. (2002). The prognostic importance of different definitions of worsening renal function in congestive heart failure. J. Card. Fail..

[21] Smith G.L., Lichtman J.H., Bracken M.B., Shlipak M.G. (2006). Renal impairment and outcomes in heart failure: systematic review and meta-analysis. J. Am. Coll. Cardiol..

[22] Brandimarte F., Vaduganathan M., Mureddu G.F. (2013). Prognostic implications of renal dysfunction in patients hospitalized with heart failure: data from the last decade of clinical investigations. Heart Fail. Rev..

[23] Damman K., Navis G., Voors A.A., Asselbergs F.W. (2007 Oct). Worsening renal function and prognosis in heart failure: systematic review and meta-analysis. J. Card. Fail..

[24] Smith G.L., Vaccarino V., Kosiborod M., Lichtman J.H. (2003). Worsening renal function: what is a clinically meaningful change in creatinine during hospitalization with heart failure. J. Card. Fail..

