# Supplementary material for: Compression wraps as adjuvant therapy in the management of acute systolic heart failure
Source: Heliyon. 2023 Aug 7;9(8):e19008. doi: 10.1016/j.heliyon.2023.e19008 (PMC10432693; doi:10.1016/j.heliyon.2023.e19008)
Supplement: Multimedia component 1 [file mmc1.pdf]

Supplementary table I: Additional information on hospitalization

	**Overall**	**Control**	**Intervention**	**p**
n	29	19	10	
Discharge weight - kg (mean (SD))	87.4 (22.2)	84.7 (20.0)	92.7 (26.1)	0.368
Oxygen on discharge - L (median [IQR])	0 [0-0]	0 [0-0]	0 [0-0]	0.478
% decrease in Sodium (mean (SD))	2.2 (2.1)	2.3 (2.1)	1.8 (2.1)	0.509
Sodium low - mg/dL (mean (SD))	133.2 (4.5)	132.8 (3.6)	133.8 (6.0)	0.591
% decrease in Potassium (mean (SD))	10.5 (8.2)	9.3 (8.6)	12.9 (7.0)	0.261
Potassium low - mg/dL (mean (SD))	3.7 (0.4)	3.7 (0.4)	3.7 (0.3)	0.953
% decrease in Chloride (mean (SD))	3.9 (3.4)	4.6 (3.6)	2.5 (2.5)	0.103
Chloride low - mEq/L (mean (SD))	97.8 (4.3)	97.3 (3.7)	98.9 (5.3)	0.337
% increase in BUN (mean (SD))	22.4 (18.6)	25.2 (18.9)	17.2 (18.0)	0.284
BUN high - mg/dL (mean (SD))	38.3 (24.7)	43.3 (28.0)	28.8 (13.1)	0.134
% increase in Creatinine (mean (SD))	17.5 (13.3)	21.1 (14.0)	10.7 (8.8)	**0.043**
Creatinine high - mg/dL (mean (SD))	1.9 (1.1)	2.1 (1.2)	1.4 (0.6)	0.101
% decrease in Magnesium (mean (SD))	9.3 (9.5)	10.9 (10.8)	6.2 (5.7)	0.215
Mg low - mg/dL (mean (SD))	1.7 (0.2)	1.7 (0.2)	1.6 (0.2)	0.134
% decrease in Phosphorus (mean (SD))	14.6 (14.5)	13.7 (15.4)	16.4 (13.0)	0.643
Phosphorus low - mg/dL (mean (SD))	3.2 (0.5)	3.1 (0.6)	3.2 (0.5)	0.873
Hypotension during admit (n (%))	13 (44.8)	8 (42.1)	5 (50.0)	0.714
Lowest MAP - mmHg (mean (SD))	71.8 (9.0)	70.1 (8.6)	75.2 (9.4)	0.150
% decrease in MAP (mean (SD))	27.3 (12.5)	28.2 (13.5)	25.5 (10.7)	0.588
Required IVF during admit (n (%))	2 (6.9)	1 (5.3)	1 (10.0)	1

BNP: brain natriuretic peptide; on admit: on admission to the hospital; BUN: blood urea nitrogen; SBP: systolic blood pressure; DBP: diastolic blood pressure; MAP: mean arterial pressure; NYHA: New York Heart Association; mg: milligram; dL: deciliter; L: liter; mEq: milliequivalent

Bolded p-values indicate statistically significant values < 0.05
