# Supplementary material for: Compression wraps as adjuvant therapy in the management of acute systolic heart failure
Source: Heliyon. 2023 Aug 7;9(8):e19008. doi: 10.1016/j.heliyon.2023.e19008 (PMC10432693; doi:10.1016/j.heliyon.2023.e19008)
Supplement: Multimedia component 2 [file mmc2.pdf]

MINNESOTA LIVING WITH HEART FAILURE® QUESTIONNAIRE

The following questions ask how much your heart failure (heart condition) affected your life during the past month (4 weeks). After each question, circle the 0, 1, 2, 3, 4 or 5 to show how much your life was affected. If a question does not apply to you, circle the 0 after that question.

**Did your heart failure prevent you from living as you wanted during the past month (4 weeks) by -**	**No**	**Very Little**				**Very Much**
1. causing swelling in your ankles or legs?	0	1	2	3	4	5
2. making you sit or lie down to rest during the day?	0	1	2	3	4	5
3. making your walking about or climbing stairs difficult?	0	1	2	3	4	5
4. making your working around the house or yard difficult?	0	1	2	3	4	5
5. making your going places away from home difficult?	0	1	2	3	4	5
6. making your sleeping well at night difficult?	0	1	2	3	4	5
7. making your relating to or doing things with your friends or family difficult?	0	1	2	3	4	5
8. making your working to earn a living difficult?	0	1	2	3	4	5
9. making your recreational pastimes, sports or hobbies difficult?	0	1	2	3	4	5
10. making your sexual activities difficult?	0	1	2	3	4	5
11. making you eat less of the foods you like?	0	1	2	3	4	5
12. making you short of breath?	0	1	2	3	4	5
13. making you tired, fatigued, or low on energy?	0	1	2	3	4	5
14. making you stay in a hospital?	0	1	2	3	4	5
15. costing you money for medical care?	0	1	2	3	4	5
16. giving you side effects from treatments?	0	1	2	3	4	5
17. making you feel you are a burden to your family or friends?	0	1	2	3	4	5
18. making you feel a loss of self-control in your life?	0	1	2	3	4	5
19. making you worry?	0	1	2	3	4	5
20. making it difficult for you to concentrate or remember things?	0	1	2	3	4	5
21. making you feel depressed?	0	1	2	3	4	5

©1986 Regents of the University of Minnesota, All rights reserved. Do not copy or reproduce without permission. LIVING WITH HEART FAILURE® is a registered trademark of the Regents of the University of Minnesota.

11/10/04
