# Supplementary material for: Compression wraps as adjuvant therapy in the management of acute systolic heart failure
Source: Heliyon. 2023 Aug 7;9(8):e19008. doi: 10.1016/j.heliyon.2023.e19008 (PMC10432693; doi:10.1016/j.heliyon.2023.e19008)
Supplement: Multimedia component 3 [file mmc3.pdf]

MINNESOTA LIVING WITH HEART FAILURE® QUESTIONNAIRE

Instructions for Data Collection and Scoring
.
1. Patients should respond to the questionnaire prior to other assessments and interactions that may bias their responses. You might tell the patient that you would like to get his or her opinion before doing your medical assessment.

2. Ample, uninterrupted time should be provided for the patient to complete the questionnaire. We recommend that the patient answer the questions without being influenced by others such as their spouse or family members. Studies show that patient proxies often have different perspectives.

3. We recommend that you use the first question to give the respondent more detailed instructions as follows.

   a. Read the introductory paragraph at the top of the questionnaire.

   b. Read the first question with the respondent – "Did your heart failure prevent you living as you wanted during the last month (4 weeks) by causing swelling in your ankles or legs?" Then tell the respondent -

   - If you did not have any ankle or leg swelling during the past month (4 weeks) you should circle the zero (0) after this question.
   - If you did have swelling that was caused by a sprained ankle or some other cause that you are sure was not related to heart failure, you should circle the zero (0) after this question.
   - If you had swelling that might be related to your heart condition, then rate how much the swelling prevented you from doing things you wanted to do or feeling the way you would like to feel. In other words, how much did the swelling affect your life? Circle either the 0, 1, 2, 3, 4 or 5 to indicate how much the swelling affected your life during the past month – zero (0) means not at all, one (1) means very little and five (5) very much.

4. Ask the patient read and respond to all 21 questions. The entire questionnaire may be read directly to the patient if one is careful not to influence responses by verbal or physical cues.

5. Check to make sure the patient has responded to each question. If a question does not apply to the patient they should circle the zero (0). Make sure there is only one answer clearly marked for each question.

Copyright University of Minnesota

6. Score the questionnaire by summating the responses to all 21 questions. In addition, a physical dimension score (items 2, 3, 4, 5, 6, 7, 12, 13 on the version sent with these instructions) and emotional dimension score (items 17, 18, 19, 20, 21) have been identified by factor analysis and may be scored by simple summation to further characterize the effect of heart failure on a patient's life.

7. Partially complete questionnaires do occur despite best efforts to minimize missing data. However, missing data can greatly bias the data and complicate analysis. To reiterate, you need to make sure the respondents understand to mark zero for any items that do not apply to them, rather than leave a blank. Whenever possible review the questionnaire before the respondent leaves to make sure there are no unanswered questions or questions with more than one answer.

8. Several methods to impute missing data are discussed in the literature.[1, 2] Multiple imputation using completed questions and perhaps other study variables to predict missing responses should be considered.[3] If a missing response is not imputed, the item will be eliminated from that person's score (the sum of responses). Since intermittently missing data can greatly affect within-person changes in scores, you might want to use the same subset of questions to represent a person at all times by omitting questions that have missing data at any point in time. We do not have any recommendations about when missing data become too extensive to render the information being collected useless.

---

[1] Fayers PM et al. Incomplete quality of life data in randomized trails: missing items. Statistics in Medicine 1998;17:679-696.
[2] Schaffer JL and Graham JW. Missing data: our view of the state of the art. Psychological Methods 2002;7:147-177.
[3] Raghunathan TE, et al. A multivariate technique for multiply imputing missing values using a sequence of regression models. Survey Methodology 2001;27:85-95.

Copyright University of Minnesota
